# Subjective assessment of mood in patients hospitalized in forensic psychiatry departments

**DOI:** 10.3389/fpsyt.2025.1728081

**Published:** 2025-12-17

**Authors:** Joanna Fojcik, Michał Górski, Rafał Skowronek, Daniel Szawarnoga, Marek Krzystanek

**Affiliations:** 1 Department of Psychiatry, Department of Neurology, Faculty of Health Sciences in Katowice, Medical University of Silesia in Katowice, Katowice, Poland; 2 Department of Chronic Diseases and Civilization Threats, Department of Occupational Medicine and Hygiene, Faculty of Public Health in Bytom, Medical University of Silesia in Katowice, Katowice, Poland; 3 Department of Forensic Medicine and Forensic Toxicology, Faculty of Medical Sciences in Katowice, Medical University of Silesia in Katowice, Katowice, Poland; 4 Department and Clinic of Psychiatric Rehabilitation in Katowice, Faculty of Medical Sciences in Katowice, Medical University of Silesia in Katowice, Katowice, Poland

**Keywords:** depression, detention, emotional state, forensic psychiatry, mood

## Abstract

**Objective:**

Subjective mood assessment is a crucial element in the diagnostic and therapeutic process in psychiatry. Assessment of subjective mood is particularly important among forensic psychiatric patients. This population is characterized by specific environmental factors—isolation, limited autonomy, and stress related to legal proceedings. This situation can significantly impact patients’ well-being and mood. The aim of this study is to discuss the specificity of subjective mood assessment in the forensic psychiatric patient population and the importance of this tool in everyday clinical practice.

**Material and methods:**

The study was conducted in forensic psychiatry departments under basic security conditions using a survey method among 112 patients. The research tool included a research questionnaire with a demographic and clinical survey and the Beck Depression Inventory.

**Results:**

The overall level of depressive symptoms among patients was relatively low. The highest symptom severity was associated with feelings of guilt, loss of interest, and pessimism. The analysis revealed a significant correlation between the length of hospitalization and the level of depression – the longer the stay, the higher the mean scores on the Beck Depression Inventory (p = 0.00001). The highest values were observed among patients hospitalized for 5 to 9 years.

**Conclusions:**

The analysis shows that patients in forensic psychiatry wards have a low level of depressive symptoms, but their severity increases with the length of stay.

## Entry

Social isolation, both voluntary and forced, has a significant impact on human emotional functioning (1). The lack of direct interactions and limited environmental stimuli can lead to low mood, feelings of loneliness, and increased anxiety. In the long term, there is an increased risk of developing depression and adjustment disorders. However, the impact of isolation depends on individual psychological resources, such as the ability to self-regulate emotions, mental resilience, and access to indirect contact (2).

Subjective mood assessment is a crucial element in the diagnostic and therapeutic process in psychiatry, allowing for the capture of the patient’s internal experience, which is often not fully visible during clinical observation (3). In the case of forensic psychiatric patients, mood assessment is particularly important because their mental functioning is closely linked to legal issues, public safety, and decisions regarding criminal liability. In this group of patients, factors such as isolation, prolonged hospitalization, limited autonomy, or tension resulting from the legal situation can significantly modify subjectively experienced mood. Analyzing this aspect allows not only for a better understanding of the patient’s mental state but also for adapting therapeutic interventions and resocialization efforts (4).

From the perspective of clinical practice, subjective mood assessment should be treated as an important component of the overall diagnostic and assessment process. It is recommended to use multidimensional procedures, e.g., combining self-reports (BDI, short mood scales, VAS), structured interviews, observational assessments, etc. Interpretation must take into account the legal context and environmental limiting factors (e.g., living in isolation) (5).

Subjective self-assessment of mood is particularly important in forensic contexts, as it reflects not only how an inmate feels but, more importantly, how they interpret their own emotions and their relationship to their behavior. This makes it an important indicator of several key psychological processes: agency, self-reflection, and readiness for change. Individuals who consciously monitor their emotions more easily learn new skills (e.g., anger management), which is one of the criteria for a positive criminological prognosis.

The medical literature lacks research on mood assessment in patients admitted to forensic psychiatry wards. Conducting such an assessment can be crucial for everyday clinical practice and help formulate recommendations regarding the need to monitor well-being and track progress in therapy and resocialization. Emotional stability is often a prerequisite for effective work on impulse control, adherence to social norms, and preparation for life in freedom.

## Material and methods

The study was conducted in forensic psychiatry wards under basic security conditions using a survey method among 112 patients. The research tool was a demographic and clinical questionnaire consisting of eight questions regarding age, gender, marital status, place of residence, level of education, professional activity, source of income, length of stay in the ward, psychiatric diagnosis, and previous suicide attempts. The Beck Depression Inventory (BDI), which allows for patient self-assessment of depressive symptoms, was also used.

Data for analysis were collected as part of a diagnostic survey study involving 112 patients (N = 112) hospitalized in basic-security forensic psychiatry wards. This sample allows for the assessment of the variables studied in the context of the specific characteristics of this patient group.

The inclusion criteria for the study group were patients hospitalized in forensic psychiatry wards with a basic level of security, male gender, stable mental state enabling the patient to freely complete the research scales, and patient consent to participate in the study. The exclusion criteria from the study group were women and patients in forensic psychiatry wards with a reinforced level of security.

The Beck Depression Inventory (BDI) is a widely recognized and widely used questionnaire measuring the severity of depressive symptoms and used in psychiatric diagnostics to assess patients’ well-being. The questionnaire consists of 21 questions, each addressing various aspects of depression, such as mood, pessimism, self-esteem, sleep disturbance, fatigue, loss of interest, suicidal thoughts, and others. Each response is scored on a scale of 0 to 3, for a maximum score of 63 points.

Based on the total number of points, the patient is assigned to one of the following depression severity categories:

- 0–11 points – no depression,
- 12–19 points – mild depression,
- 20–25 points – moderate depression,
- 26–63 points – severe depression.

Statistical analysis of the obtained results was performed using MS Excel spreadsheet and specialized Statistica software, version 13 (TIBCO Software Inc., 2017). Due to the fact that the initial analysis indicated a non-normal distribution, a nonparametric statistical method was used to verify the significance of differences between selected variables: the Kruskal- Wallis test. The adopted level of statistical significance was α = 0.05, which means that differences were considered statistically significant when the p value was lower than 0.05.

To assess differences in the severity of depressive symptoms (BDI) between categories of hospitalization length, the Kruskal- Wallis test was performed. The analysis showed a significant effect of length of stay on the level of depression: H (4, N = 112) = 8.8032; p < 0.001. The effect size was estimated using the ϵ² measure, appropriate for nonparametric tests; the value of ϵ² = 0.2318 indicates a large effect.

The study included a group of 112 men (100% – 112) hospitalized in forensic psychiatry wards. The average age of respondents was 47 years, with the youngest patient being 24 and the oldest 85, indicating a significant age range within the study population. The largest percentages were patients aged 31–40 (29% – 33) and 41–50 (28% – 31). The majority of respondents were single (56% – 63). Married individuals constituted 10% (11). 16% (18) of respondents were divorced, and 18% (20) described their status as “single.” More than half (52% – 58) of respondents lived with family. Respondents living alone constituted 39% (4). The remaining (9% - 10) respondents had previously lived in care facilities, such as a Nursing and Treatment Facility or a Social Welfare Home. Nearly half (49% - 55) of the respondents were supported by a disability pension. 19% (21) were unemployed. 2% (2) of the respondents held sheltered employment. 9% (10) were actively employed. Other sources of income were reported by 21% (24) of the respondents.

37% (41) of the study participants stayed in the hospital for 1 to 3 years. 29% (32) stayed for less than 12 months. Longer hospitalization periods of 3 to 5 years were observed in 15% (17). The same number of patients were hospitalized for 5 to 9 years and for more than 9 years (10% - 11 each).

Regarding psychiatric diagnoses, the most common (73% - 82) diagnosis was schizophrenia. Depressive disorders (5% - 6) and anxiety disorders (4% - 5) were significantly less common. Bipolar disorder (BD) was diagnosed in 4% (4) of the study participants, and 13% (15) of the patients had other psychiatric diagnoses.

The study received approval from the Bioethics Committee BNW/NWN/0052/KB/308/25. Due to the specific clinical environment, the study authors adhered to the highest scientific standards throughout the study, including the Declaration of Helsinki, which is the foundation of ethics in research involving human subjects. For the authors, the health and well-being of the study participants was their top priority.

The characteristics of the study group are presented in **Table 1**.

**Table 1:** Demographic and clinical data of the study group.

Category		N	%
Age	up to 30 years	12	11%
	31–40 years old	33	29%
	41–50 years old	31	28%
	51–60 years old	12	11%
	over 60 years old	24	21%
Sex	Man	112	100%
Marital status	State free	63	56%
	In marriage	11	10%
	After the divorce	18	16%
	Lonely	20	18%
Domicile	He lives alone	44	39%
	He lives with his family	58	52%
	Institutional care	10	9%
Education	Lack	2	2%
	Basic	20	18%
	Junior high school	16	14%
	Professional	40	36%
	Medium	29	26%
	Higher	5	4%
Professional activity/source of income	He works professionally	10	9%
	Unemployed	21	19%
	Pension	55	49%
	Work in sheltered conditions	2	2%
	Other	24	21%
Duration of stay in the ward	Up to 1 year	32	29%
	From 1–3 years	41	37%
	From 3–5 years old	17	15%
	From 5–9 years old	11	10%
	Over 9 years old	11	10%
Recognition	Schizophrenia	82	73%
	Depressive disorders	6	5%
	Anxiety disorders	5	4%
	CHAD	4	4%
	Other	15	13%
Previous suicide attempts	Yes	20	18%
	NO	92	82%

## Results

The analysis indicates that the level of depressive symptoms in the study group was generally low (**Table 2**). This is evidenced by the mean values of individual variables, which ranged between 0.21 and 0.88, with the median for all categories being 0. This means that at least half of the respondents did not report experiencing depressive symptoms in any of the analyzed areas.

**Table 2:** Beck Depression Inventory (BDI) – symptoms.

Variable	Descriptive statistics					
	N	Mean	Median	Min.	Max	Std dev
BDI - sadness	112	0.46	0	0	3	0.72
BDI - pessimism	112	0.67	0	0	3	0.82
BDI - prior failures	112	0.45	0	0	3	0.75
BDI - loss of pleasure	112	0.57	0	0	3	0.87
BDI - guilt	112	0.58	0	0	3	0.88
BDI - sense of punishment	112	0.88	0	0	3	1.17
BDI - self-dislike	112	0.46	0	0	3	0.66
BDI - self-criticism	112	0.38	0	0	3	0.75
BDI - self-destructive behavior	112	0.21	0	0	3	0.61
BDI - tearfulness	112	0.41	0	0	3	0.88
BDI - excitability	112	0.53	0	0	3	0.88
BDI - social withdrawal	112	0.59	0	0	3	0.80
BDI - indecision	112	0.67	0	0	3	0.95
BDI - loss of self-esteem	112	0.54	0	0	3	0.85
BDI - energy loss	112	0.58	0	0	3	0.87
BDI - sleep disorders	112	0.43	0	0	3	0.68
BDI - feeling of fatigue	112	0.63	0	0	3	0.78
BDI - changes in appetite	112	0.30	0	0	3	0.66
BDI - weight loss	112	0.43	0	0	3	0.80
BDI - focusing on your ailments	112	0.53	0	0	3	0.80
BDI - Loss of Interest	112	0.86	0	0	3	1.11

Among the analyzed variables, the highest mean values were obtained for feelings of punishment (0.88), loss of interest (0.86), pessimism (0.67), and indecision (0.67). The lowest mean values were observed in areas related to self-destructive behavior (0.21), tearfulness (0.41), self-criticism (0.38), and sleep disturbances (0.43).

The standard deviation of the analyzed variables ranged from 0.61 to 1.17, meaning that the results varied significantly in several categories. The greatest variability was observed for feelings of punishment (1.17), loss of interest (1.11), and indecision (0.95).

Results for the BDI symptom questions are presented in **Table 2**.

The mean global final score in the study group was 11.14, with a median of 8 points, indicating that at least half of the patients had no or very mild depression (**Table 3**). The range of scores was wide, from 0 to 46 points, suggesting significant individual differences in the severity of depressive symptoms. The standard deviation was 11.25, indicating significant variability within the study group.

**Table 3:** Beck Depression Inventory (BDI) – final score – descriptive statistics.

Variable	Descriptive statistics					
	N	Mean	Median	Minimum	Maximum	Std dev
BDI - final result	112	11.14	8	0	46	11.25

**Figure 1:**
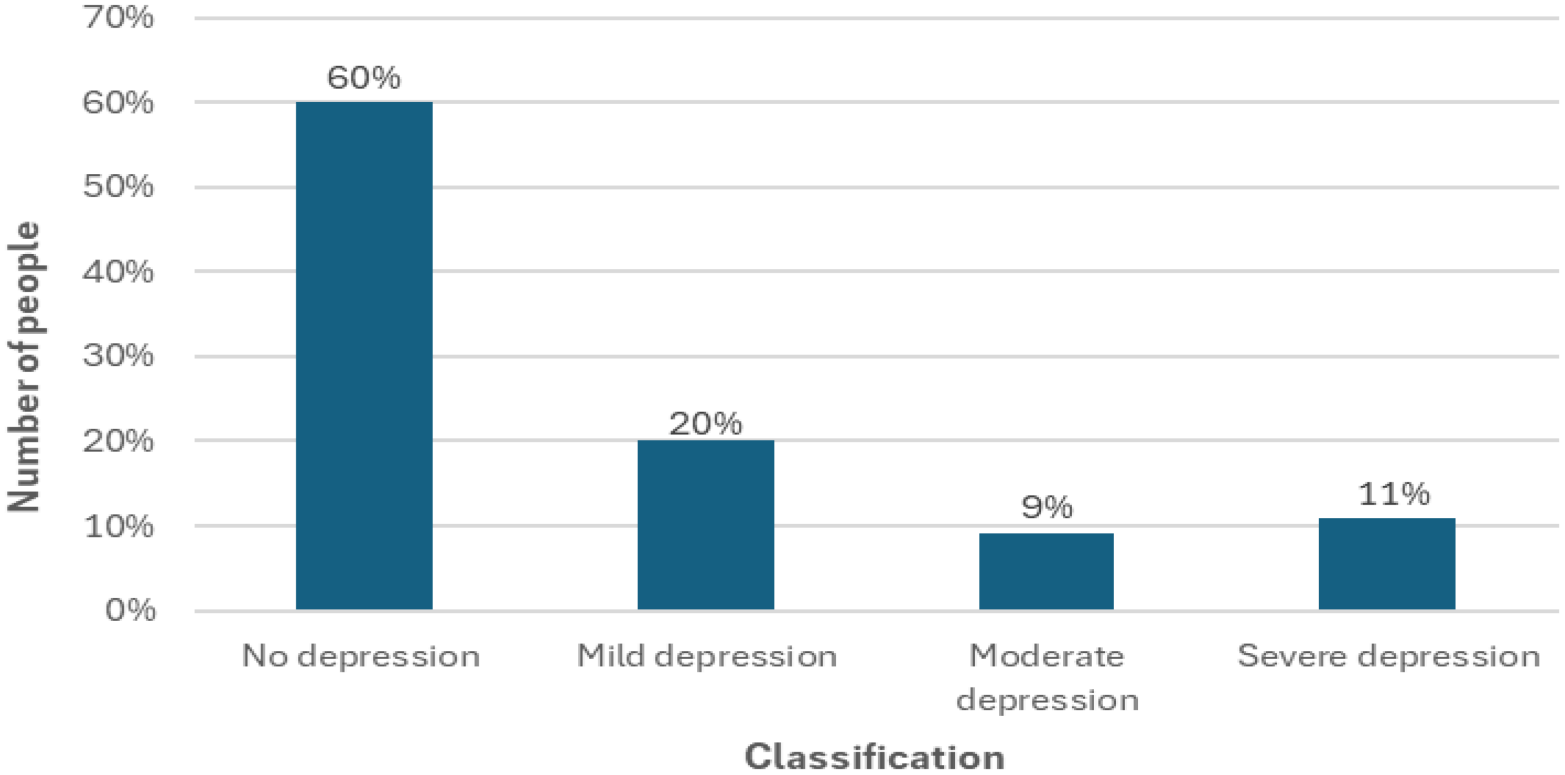
Beck Depression Inventory (BDI) – final score – depression levels (n=112).

Based on the total BDI score, patients were divided into four categories of depressive symptom severity (**Figure 1**):

1. No depression (0–11 points): 67 people (60%) – these patients did not show any significant depressive symptoms, which suggested a stable mental state.
2. Mild depression (12–19 points): 22 people (20%) – people in this group experienced moderate low mood, which may have been temporary.
3. Moderate depression (20–25 points): 10 people (9%) – symptoms in this group were more noticeable and could negatively affect the functioning of patients.
4. Severe depression (≥26 points): 13 people (12%) – these patients showed significant severity of depressive symptoms, which may have required the implementation of pharmacological treatment and psychotherapy.

Data analysis also showed a significant relationship between the length of hospitalization and the level of depression – the longer the stay, the higher the mean scores on the Beck Depression Inventory (p =0.00001).

These results suggest a nonlinear but clinically significant relationship between length of hospitalization and severity of depressive symptoms.

The results of this relationship are presented in **Table 4**.

**Table 4:** The relationship between the length of stay of patients in the forensic psychiatry ward and the final score obtained in the Beck Depression Inventory (BDI) - Kruskal- Wallis test.

Duration of stay in the ward	BDI - final score - descriptive statistics						Wallis test
	(Mean)	(N)	(Minimum)	(Maximum)	(Std.dev)	(Median)	
up to 1 year	3.97	32	0	18	5.63	2	**H (4, N = 112) =28.8032 p =0.00001**
from 1 to 3 years	11.76	41	0	38	10,11	9	
from 3 to 5 years old	13.88	17	0	35	11.10	18	
from 5 to 9 years old	25.09	11	4	46	14.76	22	
over 9 years old	11.55	11	0	26	9.53	11	

## Discussion

Mood assessment in patients hospitalized in forensic psychiatry departments plays a significant role in everyday clinical practice. Mood is a fundamental indicator of the patient’s current mental state and allows for early detection of changes that may indicate relapse or deterioration of mental health. Regular mood monitoring allows for the adjustment of pharmacological treatment and psychotherapeutic interventions to the patient’s current needs.

This assessment is particularly important in the context of safety. A depressed mood, especially when accompanied by a sense of hopelessness, may signal an increased risk of self-harm or suicidal behavior. An elevated, euphoric, or irritable mood, on the other hand, may be associated with impulsivity, agitation, and potential aggression toward others (6). In a forensic psychiatry ward, this has direct implications for protecting staff, other patients, and the patient themselves. Mood assessment is a tool for tracking progress in therapy and resocialization. Emotional stabilization is often a prerequisite for effective work on impulse control, adherence to social norms, and preparation for a potential return to an open environment (7).

In forensic psychiatry, the importance of mood assessment extends beyond therapy itself. The patient’s emotional state is one of the elements considered when issuing forensic psychiatric opinions. It can influence decisions, for example, regarding leave of absence. The relational aspect is equally important. Systematically asking the patient about their well-being and mood builds a sense of being noticed and heard. This fosters a closer therapeutic relationship and simultaneously teaches the patient self-observation and the ability to recognize and label their own emotional states. This makes mood assessment not only a diagnostic tool but also a component of the therapeutic process (8).

Pharmacological treatment in forensic psychiatry departments is also important, as its primary goal is to achieve remission of mental illness, including stabilizing patients’ mood. However, the effects depend on the type of medication, diagnosis, comorbid disorders, patient attitude, and other environmental factors. Optimal results are achieved by combining pharmacotherapy with a therapeutic approach and resocialization activities.

The results of a study on subjective mood assessment in patients admitted to forensic psychiatry wards indicate that at least half of the patients tested scored as either no depression or very mild depression, and the wide range of scores suggested significant individual differences in the severity of depressive symptoms. This study was therefore characterized by significant variability within the study group. The heterogeneity of results emphasized the need for an individualized approach to assessing patients’ mental state and implementing targeted therapeutic interventions where necessary. Similar trends were described by Smith et al. (2019) in their research on the mental well-being of people hospitalized in secure conditions, which suggests that environmental factors – such as limited autonomy, lack of privacy and the length of stay – may have a significant impact on the subjective perception of mood (9).

These data were confirmed in the conducted study. An analysis of BDI scores in the context of the length of hospitalization in forensic psychiatry showed that the length of stay significantly differentiates the final BDI score. Patients staying in the ward for less than a year have relatively low BDI scores, but these values systematically increase with length of stay. The most alarming values are found in the group hospitalized for 5 to 9 years, where the mean is 25.09 and the median is 22, indicating a significant increase in depressive symptoms. It is also worth noting a slight decrease in scores in the group of patients hospitalized for over 9 years, which may suggest adaptive mechanisms in patients who remain in the system longer.

The observed pattern—an increase in depressive symptoms in patients hospitalized in the unit from age 5 to 9, followed by a slight decrease—can be interpreted in the context of habituation, learned helplessness, or institutional adjustment. This phenomenon primarily refers to the patient’s gradual “accustoming” to the specific situations and rules of the closed environment, which can impact both their functioning and the course of therapy, as well as risk assessment. Habituation in a forensic psychiatry unit is a complex phenomenon that influences patient behavior and assessment. From a clinical and safety perspective, it can stabilize patient behavior but can also mask real risk. Therefore, this process must always be considered when assessing therapy progress and planning forensic psychiatric decisions.

Subjective mood assessment can be modified by personality factors, the presence of psychotic symptoms, and stress coping strategies (10, 11). Furthermore, the specific nature of the forensic psychiatry ward—combining treatment with legal supervision—introduces additional psychological burden not encountered in general psychiatric wards. The demonstrated correlations indicate the need for routine mood assessment in patients in forensic psychiatry wards. This will allow for the detection of worsening depressive symptoms and the implementation of psychological or pharmacological interventions, among other things, to reduce the risk of suicide. The study indicates that the BDI, despite its subjective nature, can be a useful tool for monitoring depressive symptoms in long-term detainees. Because the BDI does not require the participation of a physician, the tool can also be used by nursing staff. This is crucial given the shortage of specialized medical personnel in wards.

The study has numerous limitations. The results were derived from a group of patients treated at a single forensic psychiatric center, although they were housed in different wards. To draw general conclusions, it is necessary to compare these results with others from other centers. The results are limited to males only.

The gender limitation makes generalization difficult, especially considering possible gender differences in emotional expression and coping with isolation. It would be interesting to compare these findings with studies in women. The study used questionnaires, the reliability of which is difficult to verify and relies on the assumption of respondent truthfulness. Relying on self-assessment tools in psychotic populations raises significant issues with the validity of the obtained data. Individuals with psychotic symptoms often experience cognitive deficits that may hinder their ability to properly understand questions, assess their own emotional states, or provide consistent responses. Furthermore, the variability in insight—typical of psychotic disorders—means that their awareness of the illness and their ability to objectively self-assess may fluctuate significantly over time. As a result, self-report responses may be unstable, less consistent, and subject to distortions, limiting their diagnostic and research utility.

Future research should also explore longitudinal designs to distinguish between the influence of initial adaptation, cumulative length of stay in a closed facility, and other covariates, e.g., diagnosis, type of pharmacotherapy, and prior stays in a forensic ward.

The study also did not take into account reactive factors, such as the influence of the type of act committed on mood perception.

This study is innovative in nature. It is the first to address the value of monitoring mood in patients in forensic psychiatry departments using a simple self-assessment questionnaire. The results suggest the need to implement such monitoring into routine clinical practice in such departments. Detecting mood deterioration, especially in patients with longer stays, may necessitate the implementation of psychological and pharmacological interventions that would support emotion regulation and reduce feelings of isolation among forensic psychiatry patients.

## Conclusions

Patients in forensic psychiatry wards generally have low levels of depressive symptoms, but their severity increases with the length of stay.

## Data Availability

The original contributions presented in the study are included in the article/supplementary material. Further inquiries can be directed to the corresponding author.
